# Molecular Basis of Oxidative Stress-Induced Acute Kidney Injury, Kidney Fibrosis, Chronic Kidney Disease, and Clinical Significance of Targeting Reactive Oxygen Species-Regulated Pathways to Treat Kidney Disease

**DOI:** 10.31083/FBS38963

**Published:** 2025-09-26

**Authors:** Ramji Kandel, Priti Roy, Kamaleshwar P Singh

**Affiliations:** 1 Department of Environmental Toxicology, Texas Tech University, Lubbock, TX 79409, USA

**Keywords:** acute kidney injury (AKI), fibrosis, chronic kidney disease (CKD), oxidative stress, end-stage renal disease (ESRD), myofibroblast, cell signaling

## Abstract

Kidney disease is a growing public health problem globally. Multiple or repeated acute injuries to the kidney due to chronic exposure to toxicants promote the development of chronic kidney disease (CKD), an irreversible disease for which there is no current treatment. Renal fibrosis, characterized by glomerulosclerosis and tubulointerstitial fibrosis, is a well-known pathological stage during the progression of acute kidney injury (AKI) to CKD. Over the years, tremendous progress has been made in understanding the regulatory molecules involved in kidney fibrosis; however, there are currently no effective therapies for treating renal fibrosis. The mechanism involved in the transition of AKI to fibrosis and its progression to CKD involves various pathological changes, including cellular remodeling. At the molecular level, these pathological features are mediated by changes in the expression of genes and signaling pathways that control cellular dedifferentiation. Meanwhile, the generation of oxidative stress is a common feature of nephrotoxicants. Thus, the kidneys are highly susceptible to oxidative stress-induced injury, and accumulating evidence suggests that oxidative stress plays a causative role in the development of kidney disease. Oxidative stress has been shown to modulate various signaling pathways associated with AKI and fibrogenic changes in the kidney. Accumulating evidence suggests that targeting oxidative stress through antioxidants and/or inhibitors of reactive oxygen species (ROS)-regulated pathways holds promise for the clinical management of this disease, for which there is currently no effective therapy. This review summarizes the research development that provides a mechanistic perspective on the role of oxidative stress in regulating of target genes and signaling pathways associated with AKI and CKD. Additionally, recent reports highlighting the clinical significance of targeting oxidative stress for the treatment of CKD are discussed.

## Graphical Abstract

Schematic presentation of target genes and signaling pathways associated with ROS-induced initial stage of AKI, its transition to intermediate stage of kidney fibrosis, and finally its progression to CKD.

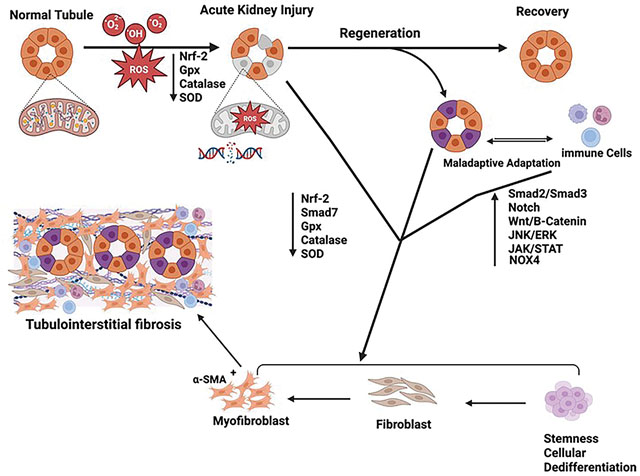

## 1. Introduction

Chronic kidney disease (CKD) is a growing public health problem globally, with rising incidence and prevalence. In the United States alone, it is estimated that approximately 808,000 people are living with end-stage renal disease (ESRD), out of which 69% on dialysis and 31% with a kidney transplant [1]. The annual mortality of ESRD patients approaches 9% per year, which is 10- to 20- fold higher than that in the general population. Multiple or repeated acute injuries to kidney due to chronic exposure to toxicants lead to the development of CKD, an irreversible disease for which there is no current treatment. Renal fibrosis, characterized by glomerulosclerosis and tubulointerstitial fibrosis, is a well-known pathological stage in kidney and is frequently observed in various forms of progressive CKD. Over the years, tremendous progress has been made in understanding the regulatory molecules of kidney fibrosis, however, currently there are no effective therapies for treatment of renal fibrosis. This review summarizes progress made in the field of kidney disease research and provides insight into (a) the role of oxidative stress in initiation of acute kidney injury (AKI) and it’s transition to fibrotic kidney that ultimately progresses to chronic kidney disease, (b) dysregulation of molecular signaling pathways associated with initiation and transition of AKI to CKD, (c) current therapeutic approaches for the treatment of kidney disease, and (d) promise and potential of target molecules dysregulated by oxidative stress during development of kidney disease.

## 2. Oxidative Stress Plays a Causative Role in AKI and Its Progression to Kidney Fibrosis and CKD

Acute kidney injury is defined as a sudden decline of kidney function along with structural and functional deterioration [2]. Kidney injury starts with inflammation and immune cell infiltration into the injury site to resolve the damage [3]. A characteristic feature of AKI is to increase serum creatinine by 0.3 mg/dL within 48 hours, 1.5 fold increase in serum creatinine levels in 7 days, or decrease in urine volume to less than or equal to 0.5 mL/kg/h for 6 hours. Based on the severity of the outcomes, kidney injury is classified as stages 1, 2 and 3 according to the kidney disease: improving global outcomes (KDIGO) staging criteria [4]. Acute kidney injury accounts for 10–15% of the hospitalized patients in the general medicine wards and exceeds up to 50% of the patients in the intensive care units [5,6]. There are several risk factors associated with the onset of acute kidney injury, including hypoperfusion, cardiorenal syndrome, nephrotoxin exposure, sepsis, significant surgery-associated fluid depletion, intrabdominal hypertension, rapidly progressive glomerulonephritis, acute interstitial nephritis and intrarenal or extrarenal obstruction [7]. AKI patients have a significantly higher risk of developing chronic kidney disease and ESRD [8]. Moreover, AKI has systemic consequences such as encephalopathy, lung injury, liver dysfunction, heart failure, intestinal and microbiota disruptions, and bone marrow and immune system effects [9,10]. There are various biomarkers for tubular injury, such as, kidney injury molecule-1 (KIM-1), interleukin-18 (IL-18), neutrophil gelatinase-associated lipocalin ( NGAL), liver-type fatty acid-binding protein (L-FABP), tissue inhibitor of metalloproteinase-2 (TIMP-2), insulin-like growth factor-binding protein 7 (IGBFP-7), and glomerular filtration (Cystatin C), which are shown to be present and increased within 6–72 hours post injury [11]. Based on the severity of the injury, damages to kidney injury might proceed to different pathophysiological outcomes. When AKI is mild, the kidney tries to recover, and the long-term outcome is full recovery of the nephron. Whereas moderate to severe AKI results in a partial to significant reduction in glomerular filtration rate (GFR) along with the increased risk of CKD, cardiovascular disease (CVD), and kidney cancer [10]. Management of damage to acute kidney injury mainly relies on discontinuation of nephrotoxic drugs, normovolemia, treatment of electrolyte disturbance and stability of hemodynamics. However, there is no treatment to support kidney function or reversal of the damage to the kidney.

In animal models, kidney injury induced by ischemia or nephrotoxins results in permanent damage to the renal microvascular and compromised renal structure and function by activating the inflammation and fibrotic signaling pathway and ultimately leading to the reduction in GFR [12,13]. Kidney fibrosis is an intermediate pathological stage during persistent AKI to CKD transition characterized by excessive extracellular matrix (ECM) deposition, thereby replacing the functional parenchymal cells. Kidney fibrosis is a common pathological outcome after acute kidney injury to repair the damage [14]. Chronic kidney disease is characterized by structural and functional damage to the kidney due to the collective damage that progressively arises from various conditions. Various risk factors facilitate the progression of kidney disease, which include acute kidney injury, genetic background, gender, ethnicity, age, low birth weight, obesity, exposure to nephrotoxins, and chronic conditions such as diabetes and hypertension [15]. CKD has an estimated prevalence between 11–13% globally and all age mortality of CKD has increased by 41.5% between 1997 and 2017 [16,17]. CKD is defined by the reduction in the function characterized by the reduction in eGFR to less than 60 mL/min or the presence of marker of kidney damage such as albuminuria hematuria or characterized by persistent structural abnormalities seen in pathological laboratory or imaging test for at least 3 months [18]. Staging of the CKD is based on eGFR (G1, G2, G3a, G3b, G4 and G5) and albuminuria (A1, A2, A3) where G5 and A3 are considered as nephrotic and kidney failure [19]. Various risk factors facilitate the progression of kidney disease, which include acute kidney injury, genetic background, gender, ethnicity, age, low birth weight, obesity, exposure to nephrotoxins, and chronic conditions such as diabetes and hypertension [15].

Kidneys are highly susceptible to oxidative stress-induced injury [20,21] and increased levels of oxidative stress have been implicated in the development of chronic kidney diseases [22,23]. Environmental nephrotoxicants (cigarette smoking, heavy metals, PAHs) are major risk factors for chronic kidney diseases. Generation of oxidative stress is the most common property of these environmental toxicants. Inflammation caused by both exogenous and endogenous factors is another important source for the increased oxidative stress in patients with kidney diseases. In addition to the exogenous sources of oxidative stress, the endogenous factors or basic characteristics of renal patients such as advanced age, diabetes and renal hypertension can also predispose individuals to increasing levels of oxidative stress compared with the general population. Molecular evidence suggests that oxidative stress due to disturbances in the formation and degradation of reactive oxygen species (ROS) is involved in the common fibrotic pathway, including renal fibrosis [24,25]. Hypoxia-inducible factor 1-alpha (HIF-1*α*)-mediated hypoxia and NADPH oxidase-derived ROS [26] have been shown to be associated with kidney fibrosis. Oxidative stress has also been shown to be involved in the fibrotic process through activation of transforming growth factor beta 1 (TGF-*β*1) [27,28]. Accumulating evidence suggests the role of oxidative stress in kidney fibrosis, however, the mechanism for oxidative stress-induced fibrosis is still poorly understood.

Oxidative stress is defined as a disturbance between the generation of reactive species and antioxidant defenses, thereby leading to potential damage [29]. Under normal physiological conditions ROS/reactive nitrogen species (RNS) is produced in different physiological processes to carry out normal cellular events. However, a supraphysiological level of ROS/RNS leads to adverse effects on cellular components such as lipids, protein, and DNA due to an inadequate counteractive antioxidant defense to maintain the physiological balance [30]. Molecular fragments with one or more unpaired electrons in molecular orbitals give rise to reactive species known as free radicals [31]. Oxygen-derived free radicals mainly account for the total free radicals in the form of ROS in living organisms. Oxygen can react with other molecules and generate secondary ROS [32]. ROS is mainly produced in mitochondria through escaped electrons during energy transduction via the mitochondrial electron transport chain [33]. Reactive nitrogen species is a molecule with one unpaired electron and is therefore considered a free radical [34]. RNS has various physiological roles, such as regulating blood pressure, neurotransmission, defense mechanism, smooth muscle relaxation and immune response regulation. RNS such as NO has a very short half-life of a few seconds, and ROS such as hydroxyl radical has a half-life of about 10^−9^ seconds [34,35]. An imbalance between antioxidant and ROS/RNS generation leads to the generation of oxidative stress. Thus, generated oxidative stress and free radicals interact with biomolecules and this results in structural and functional deterioration of the biomolecules, leading to altered cellular function and structure. Antioxidants act to scavenge or neutralize the effect of free radicals, including ROS/RNS. Antioxidants need to be taken from external sources known as exogenous or synthesized by the body as endogenous antioxidants [36]. Antioxidants act by neutralizing the effect of oxidants through enzymatic and non-enzymatic activities. Antioxidant enzymes such as superoxide dismutase, catalase, and glutathione peroxidase play a crucial role in neutralizing the effects of oxidants. Superoxide dismutase is a primary detoxification enzyme that acts by the dismutation of superoxide anion into hydrogen peroxide and molecular oxygen, reducing free radicals’ toxicity. Superoxide dismutase requires metal ions to activate, such as iron (Fe), zinc (Zn), copper (Cu), and Manganese [37–39]. Various isoforms of superoxide dismutase (SOD) have been identified, namely SOD1, SOD2, and SOD3. SOD1 requires Cu/Zn to activate SOD1, whereas SOD2 needs Mn for activation. SOD3, also known as the extracellular antioxidant. Catalase is a common antioxidant that neutralizes the generation of ROS by reducing hydrogen peroxide to water and molecular oxygen using co-factors such as iron or manganese. Catalase is mainly located in the peroxisomes; however, it is absent in mitochondria in mammalian cells except in the hearts of rats [38,40]. Similarly, glutathione peroxidase (GPx) is located in mitochondria and cytosol. GPx mainly acts by reducing hydrogen peroxide to water and molecular oxygen. Moreover, GPx is a protective barrier to the lipid peroxide by converting it to respective ethanol [38].

The following sections summarizes (a) the role of oxidative stress in initiation of AKI and their transition to fibrotic kidney that progresses ultimately to chronic kidney disease, (b) dysregulation of molecular signaling pathways associated with initiation and transition of AKI to CKD, (c) current therapeutic approaches for the treatment of kidney disease, and (d) promise and potential of target molecules dysregulated by oxidative stress during development of kidney disease.

### 2.1 Role of Oxidative Stress in Acute Kidney Injury and Fibrosis

Oxidative stress plays a role in initiating acute kidney injury by activating the pro-apoptotic pathways, eventually leading to cell death and releasing chemokines and cytokines, further exacerbating the injury by recruiting the immune cells [41]. Oxidative stress induced by cyclophosphamide leads to the peroxidation of lipids and oxidation of proteins, along with a decrease in antioxidant activity evidenced by histological damage in kidney tissue [42]. Moreover, increased levels of oxidative stress through the induction of inflammation are evidenced by the increase in critical proinflammatory cytokines in the bacterial endotoxin lipopolysaccharide (LPS) induced model of kidney injury [43,44]. Moreover, mitochondria are a major cellular organelle of interest in acute kidney injury, as shown by several studies. In human and rodent AKI, studies on the mitochondrial structure of proximal tubular epithelial cells suggests alteration in the function and ultrastructure of mitochondria [45,46]. Function of the mitochondria was shown to be altered in nephrotoxic and ischemia-reperfusion injuries in the kidneys due to fragmentation of the mitochondria. The inhibition of mitochondrial fission in the AKI rodent model ameliorated apoptosis, tubular epithelial cell damage, and injury to renal tissue [47].

Increased oxidative stress from nephrotoxicants including nicotine, arsenic, and folic acid has also been shown to alter fibrogenic genes in kidney epithelial cells [48,49]. An increase in oxidative stress was found to be correlated with inflammation and kidney fibrosis [50]. Oxidative stress has been shown to promote inflammation and release of profibrotic factors, which promote the progression of kidney fibrosis [51]. Persistent elevated levels of ROS lead to epithelial to mesenchymal transition (EMT) in diabetic kidney injury. Progression to EMT is also mediated through oxidative stress from ferroptosis and endoplasmic reticulum stress [52]. Although various mechanisms have been proposed for AKI induced by prooxidant chemicals and the progression towards fibrosis, the exact role of oxidative stress in this disease is still not fully understood.

Kidney injury proceeds through various cellular processes, including oxidative stress, inflammation, hypoxia, ferroptosis, pyroptosis, necroptosis, G2/M cell cycle arrest, and autophagy. Injury to the epithelial cells leads to cytokine secretion, which activates inflammatory response and the secretion of profibrotic cytokines [53]. Increased oxidative stress leads to damage to the cellular component, leading to cell cycle arrest in AKI, further leading to apoptosis in AKI. Oxidative stress play role in multiple pathological states, such as inducing apoptosis by activating the phosphoinositide 3-kinase (PI3K)/protein kinase B (Akt) signaling pathway [54]. This necroptosis is due to decreased mitochondrial respiration, which results in oxidative stress and inflammation [55]. Moreover, the necroptosis mediated through tumor necrosis factor alpha (TNF-*α*) is observed in AKI. A prolonged increase in the ROS in the kidney results in the activation of TGF-*β*/small mother against decapentaplegic (Smad) signaling and leads to the deposition of ECM proteins, including collagen, fibronectin, and plasminogen activator inhibitor-1 as well as reduced expression of ECM degradation factors [56].

### 2.2 Oxidative Stress-Dependent Mechanism for Progression of AKI to CKD

Mechanism for AKI to fibrosis progression involves various pathological changes. Tubular epithelial cells damage resulting in loss of tubules, cellular plasticity of EMT or partial EMT contributing to the origin of fibroblast, production of fibrogenic cytokines, activation and proliferation of myofibroblast and overproduction as well as accumulation of extracellular matrix (ECM) are some of the important biological changes associated with fibrogenic process. These pathological features of fibrosis are mediated at molecular level by events contributing to origin and activation of fibroblast through changes in expression of genes controlling cellular differentiation by EMT, mesothelial-to-mesenchymal transition (MMT), endothelial-to-mesenchymal transition (EndMT), activation of fibrogenic genes and developmental signaling pathways (TGF-*β*, wingless integration site (Wnt), Notch, Hedgehog), aberrant expression of ECM regulating genes (inactivation of matrix metalloproteinases (MMPs), activation of TIMPs). Some of the examples for the role of oxidative stress in various signaling pathways associated with AKI and its progression to CKD are summarized in Table 1 (Ref. [57–87]) and described below.

### 2.3 Oxidative Stress Modulates Signaling Pathways Associated With Acute Kidney Injury (AKI)

#### 2.3.1 Oxidative Stress and JAK/STAT Signaling in Kidney Injury

Activation of immune response Janus tyrosine kinase (JAK) and signal transducer and activator of transcription (STAT) signaling play pivotal roles in acute kidney injury. STAT acts through cytokine-mediated cell mobility, survival, differentiation, and apoptosis [88]. In AKI induced by Ischemia-reperfusion (I/R), the injury was shown to activate JAK/STAT signaling pathways. Increased levels of ROS in I/R injury result in the phosphorylation of the STAT3 and the activation of the JAK/STAT pathway, which occurs via cytokine production in I/R injury [89]. Moreover, activation of the JAK/STAT pathway in the unilateral ureteral obstruction (UUO) model of kidney injury is evidenced by the activation of STAT1 and STAT3. It leads to the macrophage polarization towards the M1 and M2 phenotype in kidney injury. Empirical evidence suggests that the activation of the JAK-STAT pathway induces apoptosis during AKI in toxicant-induced injury or Ischemia-reperfusion injury [90]. Moreover, activation of the JAK-STAT pathway is regulated by the suppressor of cytokine signaling-3 (SOCS-3) [91]. SOCS-3 enhancement is mediated by cyclic adenosine monophosphate (Epac-1) and Ras-related protein-1 (Rap-1) [92].

Oxidative stress plays a significant role in the progression of acute kidney injury through activation of the JAK/STAT pathway and the initiation of inflammation in I/R injury. Antioxidants have been shown to have reno-protective effects by scavenging ROS. For example, Ellagic acid is a naturally occurring polyphenol compound found in many fruits and vegetables and has antioxidant property. Ellagic acid has been shown to have reno-protective effect by reducing oxidative stress, inflammation, and apoptosis in renal tissue. At the molecular level, the reduced level of NOX4, which is known to be the driver of oxidative stress and triggers the JAK/STAT pathway, results in the reduced phosphorylation of JAK1, JAK2, and STAT1 and reduced damage to the renal tissue [93]. Furthermore, inhibition of JAK2 by selective inhibitor AG490 protects endothelial cells and tubular cells from oxidative stress and reduces the expression of inflammatory cytokines in cisplatin-induced kidney injury [94]. ROS generation occurs through the activation of JAK/STAT and the mitogen-activated protein kinase (MAPK) pathways, which leads to damage in DNA and proteins and promotes the mitochondrial apoptotic pathway induced by chlorpyrifos in hepatic cells [95].

#### 2.3.2 Oxidative Stress and Nrf-2 Signaling in Kidney Injury

Nuclear factor E2-related factor 2 (Nrf2) is mostly present in the cytoplasm under normal conditions. Nrf-2 is a transcription factor that is involved in platelet and erythroid development. Nrf-2 is involved in the resistance to oxidative stress and induction of drug-metabolizing pathway [96]. When there is an increased level of oxidative stress, Nrf-2 complexed with Kelch-like epichlorohydrin-associated protein 1 (KEAP1) escapes from proteasomal degradation and translocate to the nucleus and induce the expression of heme-oxygenase 1 (HO-1). HO-1 is a phase II antioxidant enzyme induced by stress and acts as an anti-inflammatory, antioxidant, antithrombotic, and anti-inflammatory. HO-1 has been shown to protect oxidative stress-induced endothelial cell injury through antioxidant activity [97,98]. Induction of the Nrf-2/HO-1 signaling pathway was shown to be induced in the AKI mice model induced by toxicant or I/R injury through the induced ROS to protect from oxidative stress-induced damage [92,99].

Nrf-2 is a natural antioxidant defense for renal tubules. Oxidative stress-induced prolonged activation of Glycogen synthase kinase (GSK) 3*β* inhibits nuclear accumulation of Nrf-2 and thereby its diminished antioxidant activity resulting in progression of acute kidney injury to the chronic kidney disease [100]. Cisplatin can induce kidney injury by increasing the levels of ROS, and in this model the diminished Nrf-2 level corresponds to the decreased levels of HO-1, Catalase, GPx, and SOD [101].

#### 2.3.3 Oxidative Stress and NF-*κ*B Signaling in Kidney Injury

Nuclear factor kappa-light-chain-enhancer of activated B cells (NF-*κ*B) is an inflammatory signal that is activated after induced inflammation. Renal injury leads to the activation of inflammatory response by secreting proinflammatory cytokines such as IL-6 and TNF-*α* through the activation of NF-*κ*B response [102]. AKI regulates NF-*κ*B signaling through cAMP-responsive element binding protein 5 (CREB5) by forkhead box Q1 (FOXQ1) mediated mechanism [103]. Moreover, in radiation-induced AKI, the NF-*κ*B signaling pathway activation occurs through the programmed cell death protein 4 (PDCD4)-mediated upregulated FGR proto-oncogene, Src family tyrosine kinase (FGR) expression [104]. Activation of the NF-*κ*B signaling pathway leads to macrophage infiltration in kidneys of the UUO model of kidney injury. In this model, the antioxidant curcumin has been shown to reduce the kidney injury by decreasing the activated NF-*κ*B, thereby further suggesting the role of oxidative stress in activation of NF-*κ*B during kidney injury [105]. The activation of immune cells occurs through co-activation of NF-*κ*B and STAT3, resulting in increased expression of inflammatory cytokines such as IL-6, TNF-*α*, and COX-2 by CCl4 treatment in mice [106]. Reduced activation of Nrf2/ARE activity enhances the activation of the NF-*κ*B pathway. NF-*κ*B/p65 antagonizes Nrf2-ARE pathway through decreased binding of CREB binding protein (CBP) and initiates recruitment of HDAC3, thus reducing the expression of the antioxidant gene [107]. In folic acid (FA)-induced AKI, an increase in NF-*κ*B and p53 expression correlates with inflammation and oxidative damage, as well as the reduced antioxidant response [108]. The selective inhibition of NF-*κ*B by PDTC reduces expression of p53 and improves renal function, suggesting interaction between NF-*κ*B and p53 in renal damage and initiation of apoptosis [108].

#### 2.3.4 Oxidative Stress and MAPK/ERK Signaling in Kidney Injury

Persistent kidney injury leads to the activation of MAPK/extracellular signal-regulated kinase (ERK) through the autophagy-mediated mechanism. This leads to the induction of transcription factor early growth response 1 (EGR1). Thus, induced EGR1 binds to the promoter of fibroblast growth factor 2 (FGF-2) [102]. Additionally, activation of the MAPK pathway through the released ATP from the Pannexin 1 channel ultimately leads to the activation of ferroptosis. Activation of MAPK/ERK signaling results in the downregulated expression of heme oxygenase in AKI [109]. Activation of the ERK/MAPK pathway resulted in COX-2 synthesis, ultimately leading to increased inflammation in LPS-induced AKI [110]. In cisplatin-induced AKI, ERK plays a crucial role in inflammation and apoptosis, shown by an increased level of phosphorylated ERK1/2 and an increase in the p53 and Bax expression along with the caspase-3 activation [111]. In LPS-induced kidney injury, oxidative stress plays a crucial role in the pathogenesis of renal damage. Increased levels of ROS were shown to induce the activation of p38 MAPK-induced vascular cell adhesion molecule-1 (VCAM-1) in Human renal mesangial cells [112]. The expression of inflammatory genes is initiated by ROS-induced activation of MAPK, which subsequently activates NF-*κ*B signaling. This activation results in NF-*κ*B’s translocation into the nucleus, which binds to the p50/p65 complex and attaches to the promoters of inflammatory genes [113].

#### 2.3.5 Oxidative Stress and PTEN Signaling in Kidney Injury

In nephrotoxic AKI, ischeamia repurfusion injury (IRI) or hypoxia reperfusion AKI, phosphatase and tensin homolog (PTEN) is shown to have protective activity during the initiation of injury. Decreased expression of PTEN promotes the process of apoptosis in AKI and results in the exacerbation of kidney damage [114]. Other reports highlighted that the induction of miR-687 through the hypoxia-inducible factor 1 (HIF-1) takes place in AKI. Thus, induced miR-687 represses the expression of PTEN, which is shown to activate cell proliferation and differentiation during AKI. Since the cells that are actively growing are more prone to damage and apoptosis. Thus, reduced expression of PTEN indirectly plays a role in apoptosis in AKI [115]. Increased levels of oxidative stress could activate PTEN in cardiac cells and have been shown to downregulate the expression of PI3K/Akt pathway, resulting in apoptosis [116]. Similarly, in cisplatin-induced acute kidney injury, downregulation of Pl3K/AKT/mTOR is seen. This downregulation of the pathway results in inflammation and apoptosis during kidney injury [117].

#### 2.3.6 Oxidative Stress and RAAS Signaling in Kidney Injury

Renin-Angiotensin-Aldosterone System (RAAS) is a hormone system that plays a vital role in regulating blood pressure, fluid volume, and electrolyte balance [118]. However, the overactivation of RAAS signaling and increased levels of the components of RAAS contribute to kidney disease including kidney fibrosis through multiple ways. For example, upregulation of Ang II through angiotensin II receptor type 1 (AT1R), activates fibrogenic TGF-*β* signaling pathways [119]. Ang II and aldosterone promotes synthesis and deposition of fibrogenic extracellular matrix such as collagen [120]. The activation of RAAS also causes progression of renal fibrosis through activation of proinflammatory response and EMT [121]. RAAS-mediated activation of inflammation further causes increased ROS production and consequently further renal damage [121]. Several studies including clinical trials has shown that RAAS inhibitors have renoprotective effect in diabetic nephropathy [122,123]. In sepsis-induced kidney injury, vasodilation leads to hypotension, which results in the activation of the RAAS enzyme. Thus, activated RAAS further leads to activating inflammatory cytokines, exacerbating kidney injury [124]. Proinflammatory cytokines, namely TNF-*α* and IL-6 produced through Ang II mediation, led to the inflammatory response, further exacerbating the renal damage [125].

### 2.4 Oxidative Stress Modulates Signaling Pathways Associated With Kidney Fibrosis

#### 2.4.1 Oxidative Stress and TGF-*β*/Smad Signaling in Kidney Fibrosis

TGF-*β*1/Smad signaling pathway is one of the key pathways during the development and progression of kidney fibrosis. There are 3 ligands of TGF-*β* which includes TGF-*β*1, TGF-*β*2, and TGF-*β*3, which have comparable activity, including cell death, differentiation, and proliferation. Binding of TGF-*β* to the receptor of TGF-*β* (commonly TGF-*β*R2) and signal transduction occurs by recruitment and phosphorylation of the TGF-*β*R1. This further phosphorylates Smad2 and Smad3 and forms complexes with the Smad4. This complex then translocates to the nucleus and promotes the expression of downstream fibrogenic genes [126]. Smad2 has protective effects, whereas Smad3 is pathogenic during kidney fibrosis development. Smad7 is also known to be the inhibitor of the TGF-*β* signaling pathway, which acts by ubiquitination of TGF-*β* R1 through the recruitment of E3 ligases SMURF1, SMURF2, NEDD4–2, or WWP1. During kidney fibrosis, loss of the inhibitory Smad (Smad7) leads to the dysregulation of the Smad signaling pathway [127]. The secreted phosphoprotein 1 (Spp1) is crucial in activating the TGF-*β*/Smad signaling pathway, which results in the differentiation of the fibroblast to myofibroblast [128]. On the other hand, bone morphogenetic proteins (BMP), a group of cytokines, have a crucial role in embryonic development and maintenance. BMP-3, a protein known to have antifibrotic properties, interacts with TGF-*β*, leading to modulation of fibroblast response to Smad3 phosphorylation and inhibits the development of fibrosis. Loss of BMP-3 is commonly observed in fibrotic disease [129].

Increased oxidative stress plays a crucial role in the induction of TGF-*β*, which results in the onset of renal disease [130]. In renal tubular epithelial cells, treatment with the TGF-*β*1 was shown to induce ROS production and induced the process of EMT. Activation of Smad2 by phosphorylation takes place through the ROS, evidenced by the reversal of TGF-*β*1–induced phosphorylation of Smad2 by antioxidants [131]. Activation of Smad2 takes place through the induction of ERK activity. Thus, induced ERK also binds to the collagen promoter region and aids the production of ECM in renal mesangial cells [132]. The endothelial-to-mesenchymal transition (EndMT) induced by H_2_O_2_ through activation of Smad3 involving TGF-*β*1 activated activin receptor-like kinase 5 (ALK5) results in the expression of fibrotic marker. It diminishes the expression of endothelial markers [133].

Moreover, TGF-*β*1 also induces the differentiation of cardiac fibroblast to myofibroblast. This process is driven by the upregulated expression of NOX4, which facilitates the production of ROS and the expression of alpha-smooth muscle actin (*α*-SMA). Nox4 elicits prolonged phosphorylation and activation of Smad 2/3 [134]. In the kidneys of diabetic mice, a reduction in the cytoplasmic antioxidant NAD(P)H quinone oxidoreductase 1 (NQO1) was observed, accompanied by an increase in proinflammatory cytokines and collagen deposition. Overexpression of NQO1 through an adeno-associated virus led to decreased ROS levels, reduced ECM deposition, and inhibition of TGF-*β*/Smad signaling, resulting in an improved fibrotic outcome in the diabetic mouse kidney. This suggests that elevated ROS levels and fibrosis in diabetic mice kidneys are driven by activation of the TGF-*β*/Smad pathway [135].

#### 2.4.2 Oxidative Stress and JAK/STAT Signaling in Kidney Fibrosis

The Janus tyrosine kinase (JAK)-STAT signaling Pathway is crucial for cellular function, including tissue repair, hematopoiesis, immune function, inflammatory response, and apoptosis [46]. JAK2/STAT3 activation occurs through the binding of the TGF-*β*1 and PDGFR-*β*. TGF mediates the activation of JAK-2 leading to phosphorylation of STAT3 and translocation into the nucleus [136]. Activation of JAK-2 regulated pathway in human diabetic nephropathy was observed and this process leads to the development of glomerulosclerosis, fibrosis ultimately leading to renal failure. However, a similar kind of JAK-2-mediated response was not observed in the mice model of diabetic nephropathy, resulting in less severe tubulointerstitial fibrosis [137]. Activating the JAK/STAT pathway through angiotensin II promotes the expression of extracellular matrix components such as TGF-*β*, collagen-IV and fibronectin and promotes the development of renal fibrosis [137]. The activation of STAT3 plays a role in the presence of TGF-*β* in the UUO model of kidney fibrosis. Active STAT3 is shown to significantly increase in interstitial cells. Presumably myofibroblasts play a role in the production of ECM [138]. Inhibition of the STAT3 using specific inhibitor S3I-201 resulted in inhibiting fibronectin and *α*-SMA expression, implying the inhibition of myofibroblast proliferation. Moreover, this inhibition of STAT3 results in the deposition of ECM and the recruitment of proinflammatory cytokines in the UUO model of kidney fibrosis, implying that STAT3 plays a role in kidney fibrosis by recruiting inflammatory response and activation of fibroblast [139].

An increased level of intracellular ROS is shown to activate the JAK/STAT pathway through phosphorylation [57,140]. Thus, phosphorylated and activated JAK/STAT translocate to the nucleus, resulting in activation of proinflammatory factors such as intercellular adhesion molecule 1 (ICAM-1) and IL-6 along with the pro-fibrotic genes [57]. In diabetic kidney disease, STAT1 activation was observed, and the expression of the Forkhead box O1 (FoxO1) was decreased. Overexpression of FoxO1 results in less fibrosis development and inhibits tubular cells through the antioxidant activity of FoxO1 [141].

#### 2.4.3 Oxidative Stress and Wnt/*β*-Catenin Signaling in Kidney Fibrosis

Wnt/*β*-catenin is an evolutionary pathway that takes part in organ development and repair. It is a canonical pathway that, under normal conditions, remains in inactive states. This inactive state of Wnt signaling is maintained by phosphorylating *β*-catenin by APC-Axin-GSK-3*β* complex, ultimately leading to the degradation of *β*-catenin. During AKI progressing toward fibrotic condition, Wnt binds to the cellular surface receptor, disrupting the phosphorylation and degradation of *β*-catenin. This results in the accumulation of the *β*-catenin in the cytoplasm, which eventually passes into the nucleus. *β*-catenin interacts with the transcription factor T cell factor/lymphoid enhancer factor (TCF/LEF), eventually leading to increased expression of *β*-catenin target genes such as MMP-7 [142]. MMP-7 acts as a surrogate marker of kidney injury and is involved in the pathogenesis of kidney fibrosis by activating the *β*-catenin signaling pathways. The pathogenic mediation of the MMP-7 is due to promoting EMT and compromising integrity by altering the adherence receptor [142]. Indirect regulation of *β*-catenin activity and stability by NF-*κ*B in kidney fibrosis takes place by promoting the degradation of *β*-catenin. Translocation of *β*-catenin enhanced by inhibitor of nuclear factor kappa-B kinase subunit alpha (IKK*α*) further enhances the expression of fibrosis-associated genes such as fibronectin and fibronectin, *α*-SMA, and cyclin D1 [143]. Moreover, the activation of the Wnt/*β*-catenin leads to the activation of fibroblast to myofibroblast, proliferation, and differentiation of myofibroblast through the TGF-*β*/Smad dependent pathway leading to fibrosis [130]. The binding of the *β*-catenin and p65 to the fibronectin promoter, along with the decreased expression, results in the activation of fibronectin [144]. Activation of the Wnt/*β*-catenin leads to the increased expression of genes in the Renin-angiotensin system (RAS), which promotes the initiation and progression of kidney fibrosis in Glycogen storage disease type Ia (GSD-Ia) mice [145].

Elevated levels of oxidative stress drive the activation of the Wnt/*β*-catenin signaling pathway. This is demonstrated by improved renal injury, oxidative stress, inflammation reduction and restoration of *β*-catenin and Wnt-4 expression following geraniol treatment in cyclosporin A-induced renal injury model [58]. Activation of the *β*-catenin signaling occurs through the induction of RAAS, which results in the oxidative, inflammatory, and fibrosis-associated pathways accompanied by oxidative stress [146]. In advanced oxidation protein products (AOPPs)-induced kidney injury it results in the Nox (NADPH oxidase) induced generation of ROS. Thus, the ROS induces NF-*κ*B and downstream targets such as Wnt/*β*-Catenin, resulting in podocyte injury. This results in the subsequent phenotype alteration by expressing desmin, fibronectin, MMP9, Snail1, and proteinuria [59]. Activation of Wnt3*α*/*β*-catenin further triggers ROS production and promotes chronic inflammation [60].

#### 2.4.4 Oxidative Stress and Notch Signaling in Kidney Fibrosis

Notch signaling is a conserved pathway mainly involved in cell-cell communication for various functions, including cell fate decision, cell lineage specification, and stabilization. At the cellular level, there are four Notch receptors, namely Notch1, Notch2, Notch3, and Notch4 [139]. Notch receptors have several ligands, such as Jagged1 (Jag1), Jagged2 (Jag2), Delta-like 1 (Dll 1), Delta-like 3 (Dll 3) and Delta-like 4 (Dll 4). Notch signaling has a vital role in the development of the kidney and mutation in the notch receptor leads to developmental abnormalities in the kidney [147]. The notch signaling pathway is involved in renal fibrosis, evidenced by increased expression of Notch1 associated with tubulointerstitial fibrosis in human renal biopsies and the decreased estimated GFR. The expression of Notch2 was observed to have an inverse relationship with the level of tubulointerstitial fibrosis, suggesting Notch1 and Notch2 have distinct roles in disease progression [148]. Additionally, Notch3 seems to have a role in the progression of renal fibrosis, which is evidenced by the knockout of Notch3 shown to have protective effects on fibrosis development with the smaller number of alpha SMA staining cells. Notch3 promotes fibrosis through the recruitment of the inflammatory cells [149].

An increased level of ROS activates Nrf2, which furthers the Notch signaling pathway for the proliferation of the airway epithelium [9]. Upregulating Notch1 mediated oxidative stress and inflammatory response generation in uric acid treated in human umbilical vein endothelial cells (HUVEC) [150]. Moreover, in HUVEC cells, Notch signaling was associated with the H_2_O_2_-induced oxidative damage, and inhibition of the Notch signaling pathway was shown to be protective by manifesting improved cell viability and reduced apoptotic marker [151]. Conversely, other reports highlight the antioxidant role of Notch1 through interaction with Nrf2. The emodin, known to cause nephrotoxicity, induced oxidative stress and ferroptosis by downregulating the activity of Notch1/Nrf2/glutathione peroxidase 4 (GPX4) [152]. Activation of Notch signaling results in the generation of oxidative stress and progression toward pulmonary fibrosis, suggesting a crucial role of oxidative stress and Notch signaling in pulmonary fibrosis [139,153]. Oxidative stress-induced activation of Notch1/ADAM7 through *γ*-secretase and TGF-*β* activation leads to uterine fibrosis in mice exposed to polystyrene microplastics (PS-MP). Inhibition of the TLR4/NOX2 results in reduced ROS and consequential outcome of fibrosis through the inhibition of Notch1 and TGF-*β* activation [154].

#### 2.4.5 Oxidative Stress and MAPK Signaling in Kidney Fibrosis

MAPK signaling pathway consists of a group of serine-threonine proteins, which include MAPK, extracellular signal-regulated kinase, and c-JUN N-terminal kinases (JNK). MAPK signaling is involved in response to cellular stress by extracellular stimuli, neurotransmitters, and cytokines, resulting in cell proliferation, differentiation, metastasis, and apoptosis. MAPK signaling pathway is shown to be activated in both acute kidney injury and chronic kidney disease. It works alongside the TGF-*β*1; activation of MAPK occurs in the presence of TGF-*β*1 while activated MAPK further promotes expression of TGF-*β*1 in different kidney cells during the development of kidney fibrosis [155]. TGF-induced activation of p38 MAPK induces EMT, promoting ECM deposition by inhibiting the degradation of ECM. Inhibition of the TGF-*β*1/p38 MAPK pathway by its selective inhibition with SB203580 has been shown to reduce the level of fibrosis [156]. Activation of p38 MAPK regulates the expression of PDZK1 through increased mitochondrial ROS, resulting in EMT and renal fibrosis. Inhibiting p38 MAPK or Pl3K/AKT restored the expression and subsequently alleviated the progression of kidney fibrosis in the UUO or Adenine-induced kidney injury model [157].

Previous reports suggest that ROS and particularly the mitochondrial ROS regulate TGF-*β*/MAPK signaling during kidney disease development. For example, ROS production induced by NOX4 activation and mediated by TGF-*β*1 results in the activation of the p38/MAPK pathway causingmitochondrial damage. This further leads to the activation of mitophagy and consequently the EMT and fibrogenic process [158]. In liver fibrosis, the activation of hepatocyte stem cells is shown to be activated by ROS through a p38-mediated mechanism [159]. Accumulation of mitochondrial ROS results in the activation of the MAPK signaling pathway, which contributes to inflammation and fibrosis in idiopathic pulmonary fibrosis. This process is governed by the translocation of activated p38 MPAK to the nucleus and regulates the expression of genes involved in inflammation, cell proliferation, and ECM [160]. Inhibition of Nox1/4 has been shown to be reno-protective by inhibiting oxidative stress and activation of ERK1/2 and MAPK in diabetic renal injury [161]. Taken together, these reports suggest that ROS play a critical role in activation of TGF-*β*/MAPK signaling during myofibroblast activation and proliferation in kidney fibrosis as well as its progression to CKD.

#### 2.4.6 Oxidative Stress and Sonic Hedgehog Pathway in Kidney Fibrosis

The Sonic hedgehog signaling pathway (Shh) is involved during organ development. Shh is a morphogen secreted extracellularly. During development of the kidney, Shh plays a regulatory role in cell cycle regulation and tissue patterning [162]. In a normal kidney, Shh remains inactive and has base-level expression in the kidney. Zhou *et al*. [163] show that the expression of Shh is induced in different animal models, including UUO, IRI, adriamycin (ADR) nephropathy, 5/6 nephrectomy, and human CKD samples. Moreover, the induced expression of Shh promotes interstitial fibroblast proliferation and transition to myofibroblast. This, in turn, results in the development of fibrosis. Interestingly, the inhibition of Shh controls the proliferation of fibroblasts and alleviates kidney fibrosis [163].

Oxidative stress-induced injury has been shown to play a crucial role in the increased expression of SHH protein without mRNA expression alteration. This secretion of SHH from the injured cell is a reparative response to oxidative damage and TGF-*β*–induced remodeling in idiopathic lung disease [164]. A recent report by Kim *et al*. [165] demonstrated that hyperglycemia-induced Shh and TGF-*β* pathway activation leads to the fibrogenic phenotype in renal proximal tubular epithelial cell line.

## 3. Oxidative Stress-Dependent Programmed Cell Death as Emerging Mechanism for AKI and CKD

In addition to apoptosis, additional programmed cell death processes such as necroptosis and ferroptosis have also emerged as mechanisms for oxidative stress-induced various forms of kidney disease including AKI and CKD [166]. Mitochondrial ROS (mtROS) can induce necroptosis through autophosphorylation of receptor-interacting serine-threonine kinase 1 (RIPK1) which is required for other factors to form necrosome for necroptosis [167]. Necroptosis has been shown to be involved in the animal model of AKI [168]. Free radicals can also react with cellular lipid components resulting in oxidized form of lipids a process known as “lipid peroxidation”. Iron-dependent lipid peroxidation is one of the known mechanisms for ferroptosis. Role of mitochondria in ferroptosis has also been reported [169]. There are conflicting reports on the role of ferroptosis in kidney disease. For example, Martin-Sanchez *et al*. [170] demonstrated that ferroptosis, but not necroptosis, is the major cause of cell death in folic acid-induced AKI. However, a rat I/R kidney injury model revealed that miR-182–5p and miR-378a-3p could bind GPX4 and SLC7A11 mRNAs to inhibit their expression, which in turn activated ferroptosis to reduce I/R kidney injury suggesting a protective effect of ferroptosis in AKI [171]. Mitochondria-specific autophagy or mitophagy is an autophagic response that selectively targets impaired, permeabilized, and dysfunctional mitochondria thereby eliminating their harmful effects in the cell such as inflammation. Damaged or dysfunctional mitochondria has been shown to cause oxidative stress associated with various forms of kidney diseases. Mitophagy mediated recycling of damaged mitochondrial components thereby is considered as a cytoprotective process in AKI and CKD [172]. Contrary to these protective effects of mitophagy in kidney disease, another study suggests that excessive mitophagy can actually cause kidney disease. For example, dynamin-related protein 1 (Drp1)-dependent induction of mitophagy has been shown to be associated with renal I/R injury [173].

These reports suggest that cellular context-dependent and mitochondria-dependent multiple mechanisms exist for programmed cell death during AKI and its progression to other forms of kidney disease.

## 4. Current and Emerging (Clinical Trials) Therapeutic Approaches for Kidney Disease

In a clinical trial Empagliflozin, a known sodium glucose cotransporter 2 (SGLT2) inhibitor, has shown promise in reducing the progression of kidney disease [174]. Another SGLT2 inhibitor, Dapagliflozin, has also demonstrated effective results in clinical trials, indicating a reduced risk of developing chronic kidney disease and death from renal failure, regardless of the presence of diabetes [175]. Similarly, canagliflozin has been associated with a reduced risk of kidney failure in patients with diabetic kidney disease [176]. Reno-protective effects of SGLT2 inhibitors are thought to be mediated by their ability to reduce renal oxidative stress and ROS signaling associated with kidney disease [177,178]. Additionally, the diabetes drug semaglutide acts as a GLP-1 receptor agonist and has been shown to have protective effects on kidney function in patients with both kidney disease and diabetes [179]. Its renoprotective effects are primarily attributed to its capacity to reduce inflammation, oxidative stress, and fibrosis [180,181]. Renoprotective effect of the nonsteroidal selective mineralocorticoid receptor antagonist Finerenone was evaluated in patients with advanced CKD and type 2 diabetes. It showed a reduction in the progression of chronic kidney disease and improved cardiovascular outcomes [182]. On the other hand, the endothelin and angiotensin antagonist, Sparsentan, has been shown to have protective effects on the kidneys by reducing proteinuria and preserving kidney function in patients with IgA nephropathy [183].

In addition to the above-mentioned clinical trials with drugs for kidney disease, several other Food and Drug Administration (FDA)-approved drugs for diabetes have also shown promising results in inhibiting or slowing the progression of kidney disease in animal models. For example, linagliptin, a dipeptidyl peptidase-4 (DPP-4) inhibitor and FDA-approved glucose-lowering drug for type 2 diabetes, have shown reno-protective effects in rodent models of both non-diabetic as well as diabetic kidney disease [184]. Empagliflozin, a SGLT2 inhibitor, significantly reduced kidney fibrosis by inhibition of EMT in diabetic mice model [185]. Similarly, the renoprotective effects of inhibitors of angiotensin-converting enzyme (ACEi), angiotensin receptor blocker (ARB), and Mineralocorticoid Receptor Antagonists (MRAs) have also been shown in animal models [186]. Although these therapeutics have shown promising results in slowing the progression of kidney disease, as of now there is no drugs available to cure kidney fibrosis and CKD.

## 5. Clinical Relevance of Targeting Oxidative Stress to Prevent Progression of Kidney Disease

Considering the significant causative role of oxidative stress in AKI and its progression to CKD, numerous clinically relevant therapeutic strategies targeting oxidative stress have been developed for the treatment of this disease. The aims of these antioxidant-based therapies are to either reduce the levels of ROS and its adverse effects in kidney or enhance the levels of antioxidants that can help in slowing the progression of kidney damage to CKD by mitigating oxidative stress. For example, N-acetyl cysteine has been shown to protect against diabetic nephropathy by alleviating mitochondrial damage and ferroptosis through the activation of the SIRT3-SOD2-Gpx4 signaling pathway in beagle dogs with diabetic nephropathy induced via streptozotocin (STZ) [187]. N-acetylcysteine has demonstrated protective effects against kidney injury during AKI caused by contrast-induced nephrotoxicity, emphasizing the role of oxidative stress in renal damage [188]. Metformin is an oral antihyperglycemic drug that targets mitochondria and inhibits complex I [189]. Metformin was shown to prevent the progression of chronic kidney disease in an adenine-enriched diet model of chronic kidney disease in rats [190]. The SGLT2 inhibitor empagliflozin has been shown to alleviate diabetic nephropathy by restoring the expression of peroxiredoxin 3 (Prdx3) and reducing the levels of ROS and mitochondrial ROS in a diabetic (db/db) mouse model [191]. Empagliflozin restores SIRT3 levels, thereby inhibiting kidney fibrosis through the prevention of EMT [185]. Atorvastatin is a cholesterol-lowering drug that inhibits 3-hydroxy-3-methylglutaryl-coenzyme A (HMG-CoA) reductase. It has been shown to protect against kidney fibrosis in both diabetic and hypertensive rat models of renal injury. This protective effect is attributed to its ability to reduce oxidative stress and inflammation [61,192]. Resveratrol is a naturally occurring flavonoid recognized for its ability to scavenge reactive oxygen species (ROS). Research has demonstrated that it protects against the progression from AKI to CKD by reducing tubular damage, restoring oxidative balance, repairing mitochondrial damage, and modulating profibrotic signaling during ischemia-reperfusion injury [193]. Green tea polyphenol epigallocatechin gallate (EGCG), known for its antioxidant activity, has been shown to restore fibrogenic changes in kidney epithelial cells [194]. Similarly, EGCG exhibits a renoprotective effect on diabetic nephropathy by inhibiting ROS-induced TGF-B upregulation and preventing apoptosis in STZ-induced diabetic rats [195]. Autophosphorylation of apoptosis signal-regulating kinase 1 (ASK1) triggered by ROS leads to inflammatory response in diabetic kidney disease (DKD) [196]. ASK1 inhibitors, GS-444217 and GS-4997 have been shown to have a renoprotective effect in several in vivo animal models of kidney disease and are currently in clinical trials for treatment of DKD [197].

## 6. Conclusion and Future Direction

As mentioned above, over the years, tremendous progress has been made in understanding the molecular regulators of kidney fibrosis. However, currently there are no effective therapies for treatment of renal fibrosis, a commonly observed pathological stage during CKD development. Accumulating evidence suggests the role of oxidative stress in regulation of genes and signaling pathways associated with CKD. However, further research is needed to elucidate the precise mechanism underlying oxidative stress-induced kidney fibrosis and CKD. Recent reports suggest that several profibrotic genes and pathways are altered in kidney fibrosis through epigenetic dysregulation. Therefore, future research on identifications of epigenetic modifications and the target genes affected by those modifications can further explain the molecular basis for fibroblast activation and kidney fibrosis and CKD. As there are no effective therapeutics for the cure of kidney fibrosis and CKD, identification of oxidative stress-triggered and epigenetically regulated target molecules associated with CKD will be of tremendous significance in clinical management of this disease.

## Figures and Tables

**Table 1.** Signaling pathways dysregulated by oxidative stress in kidney disease.

Signaling pathway	Altered proteins	Effect of oxidative stress	Models of kidney injury	Reference

TGF-*β*/Smad	Increased Nox-4 and p-Smad-3 expression	Smad-3 activation of NOX-4	Streptozotocin (STZ) induced diabetic kidney injury models; Ischeamia repurfusion injury (IRI) models in C57BL/6	[62,63]
TGF-*β*/Smad	Increased expression of Smad-2/Smad-3	Increased collagen deposition	Male long Evan rats with nephrectomy injected with FCA and a 1% salt (NaCl) solution to induce hypertension	[64]
TGF-*β*/Smad	Increased expression of p-Smad	Activation of Smad-3 signaling Increased Nox4 expression, *α*-SMA, Fibronectin and collagen	Male C57BL/6 mice as UUO and Folic acid induced kidney injury model	[65]
TGF-*β*/Smad	Increased expression of p53 and smad3	Binding of p53 and Smad3 Increased PAI and fibronectin expression	UUO model of kidney fibrosis and HK-2 human proximal tubular epithelial cells treated with TGF-*β*1	[66]
TGF-*β*/Smad	Increased expression of NUAK1	Induce YAP and Smad signaling and promoting expression of fibrogenic genes	Wild-type C57BL/6 mice of folic acid and UUO model of kidney fibrosis; normal rat kidney interstitial fibroblasts (NRK49F)	[67]
TGF-*β*/Smad	Decreased expression of KP1 Increased expression of Smad2/3 and p-Smad2/3	Increased fibrotic lesion Increased fibronectin, collagen I and *α*-SMA expression	Male BALB/c mice UIRI or UUO model; Normal rat kidney interstitial fibroblasts (NRK-49F) and human proximal tubular epithelial cells (HKC-8) after 24 h of TGF-*β*1 treatment	[68]
TGF-*β*/Smad	Increased ratio of p-Smad2/3 to Smad2/3	Increased expression of slug, Vimentin, CTGF and *α*-SMA	UUO model of kidney injury in Male swiss albino mice	[69]
TGF-*β*/Smad	Decreased expression of Smad7	Increased activation of TGF-*β*1/Smad signaling Increased deposition of collagen Decreased Nrf2 expression	C56BL/6 male mice injected with aristolochic acid (AA) *i.p.* injections at 5 mg/kg body weight	[70]
Notch signaling	Increased expression of Notchl and HESl	Activation of Notch/Hes-1 signaling Increased expression of cleaved caspase 3 Decreased HO-1 expression	Sprague-Dawley rats injected with Gentamicin (GM) *i.p.* at 100 mg/kg body weight	[71]
Notch signaling	Increased expression of Notchl, Jagged-1	Activation of Notch/Jagged pathway Increased TGF-*β* R1, collagen I and *α*-SMA expression	Male Sprague-Dawley rats were fed a mixture of adenine (0.1 g/kg) and potassium oxonate (1.5 g/kg) daily for 3 weeks; Rat renal interstitial fibroblasts (NRK-49F) were exposed to uric acid (800 μmol/L) for 36 hrs	[72]
Nrf-2 signaling	Decreased expression of Nrf-2	Decreased expression of GPx4 Morphological damage to mitochondria Increased Renal tubular injury and ECM deposition	Diabetes was induced by injecting glucose to male C57BL/6 *i.p.* injections of 50 mg/kg/day for 5 days HK-2 cells were treated with high levels of glucose	[61]
Nrf-2 signaling	Decreased expression of Nrf-2	Increased expression of fibrogenic signaling including Wnt-1, *β*-catenin, TGF-*β*1, and p-Samd3	High fat diet induced nephrotoxicity in Kunming mice	[73]
Nrf-2 signaling	Decreased expression of Nrf-2 Increased expression of NF-κB, TXNIP and NLRP3	Increased collagen deposition and inflammatory response Increase in tubular injury	STZ-induced Diabetic Nephritis in male Sprague Dawley rats	[74]
Nrf-2 signaling	Decreased expression of Nrf-2	Increased TGF-*β*, collagen I, collagen IV and Bax expression	STZ-induced-Diabetic Nephritis in male C57BL/6 mice	[75]
Nrf-2 signaling	Increased cytosolic Nrf-2, HO-1 and NF-κB-p65 expression Decreased expression of nuclear Nrf-2, SOD2 and catalase protein	Increased collagen I and fibronectin production and deposition	UUO models of kidney injury in C57BL/6 mice	[76]
Nrf-2 signaling	Decreased Nrf-2 protein translocation and activity	Induced fibrogenic signaling including TGF-*β* mediated pathway Increased ratio of Bax/Bc1-2 and caspase 3 and PARP expression	UUO models of kidney injury in Female Wistar rats	[77]
JAK/STAT signaling	Increased JAK2, STAT3 andNox4 protein expression	Increased collagen deposition and tubulointerestitial fibrosis development	Hypertensive renal injury model in Male Sprague-Dawley rats	[78]
JAK/STAT signaling	Decreased expression of suppressor of cytokine signaling (SOCS1), activation of STAT1/3	Increased Renal damage and mesangial cell expansion	Diabetes induced by STZ injection in ApoE knockout mice and WT C57BL/6J male mice; Mouse mesangial cells and Mouse kidney proximal tubular epithelial cells	[79]
JAK/STAT signaling	Increased expression of p-JAK2, P-STAT3 and SOCS1 proteins	Increased expression of TGF-*β*1, Collagen-I, Bax and fibronectin	Male inbred Sprague-Dawley (SD) rats fed with high-glucose-high-fat diet and injected with STZ solution; HK-2 cell	[57]
JAK/STAT signaling	Increased NOX4 expression	Increased collagen deposition Increase oxidative DNA damage in glomerular and tubulointerstitial region	Diabetes induced by STZ in ApoE deficient mice	[80]
JAK/STAT signaling	Increased JAK-2, STAT1 and STAT3 mRNA expression	Increase in KIM1 release	Human renal PTEC cells exposed to glycated albumin (AGE-BSA) and high glucose	[81]
Wnt/*β*-catenin Signaling	Increase in *β*-catenin, Wnt4 and TGF-*β*	Decreased expression of E-Cadherin, Smad7 and PPAR*γ* Increased tubular necrosis and protenacious casts Increase in TGF-*β*1, caspase3 and p-Smad3 expression	Male Wister rats were exposed to cyclosporine A (CsA) via gastric gavage	[58]
Wnt/*β*-catenin Signaling	Increased B-Catenin, Nox2, Wntl, Wnt2, Wnt 7a and Wnt 9a	Increased active B-catenin, Fibronectin, Snaill and *α*-SMA	Male C57BL/6 mice were induced podocyte injury by injecting iv injection of advanced oxidation protein product (AOPP)	[59]
Wnt/*β*-catenin Signaling	Increased Wnt 3a and B-catenin expression	Apoptotic cell death, endoplasmic reticulum stress and mitochondrial damage	UUO model at the Male Sprague Dawley rats; HK-2 cell	[60]
MAPK Signaling	Increased p-p38, p-ERK expression	Activation of Smad3 Increased Nox4 expression, Alpha-SMA, Fibronectin and collagen	Male C57BL/6 mice as UUO and Folic acid induced model	[65]
MAPK Signaling	Increased p-ERK1/2, p-p38 and p-JNK expression	Increased fibrotic lesion Increased fibronectin, collagen I and *α*-SMA expression	Male BALB/c mice UIRI orUUO model; Normal rat kidney interstitial fibroblasts (NRK-49F) and human proximal tubular epithelial cells (HKC-8) after 24 h of TGF-*β*1 treatment	[68]
Nrf-2 signaling	Increased p-JNK, p-p38 and p-ERK expression	Increased TGF-*β*, collagen I, collagen IV and Bax expression	STZ-induced-Diabetic Nephritis in male C57BL/6 mice	[75]
MAPK and NF-κB signaling	Increased expression of p38	Decreased expression of HO-1 and Nrf-2 Increased *α*-SMA and pSmad2/3	STZ induced Diabetic mice in male adult Sprague-Dawley (SD) rats	[82]
MAPK Signaling	Increase in MAPK, ERK and p38 mRNA level	Increased expression of fibronectin, *α*-SMA, and MMP-2	Male ICR mice injected with methylglyoxal-derived hydroimidazolone-l (MG-Hl); HK-2 cell	[83]
MAPK Signaling	Increase in p-ERK1/2, p-PKC*α* and p-p38 expression	Increased NOX4, Fibronectin and *α*-SMA expression Increase in PCNA, CyclinDl STAT3 and p-STAT3 expression	UUO model in Male BALB/c mice; 5/6 nephrectomy (5/6NX) in male CDl mice; HK-2 cell; NRK-49F cells	[84]
MAPK Signaling and NF-κB signaling	Increase in p-ERK1/2, p-38 and MAPK	Increase inNOX4 and HO-1 expression Increase in BAX, cleaved caspase3, TGF-*β*1, CTGF and *α*-SMA expression	UUO model of kidney fibrosis in C57BL/6J mice	[85]
MAPK Signaling	Increase in p38 and MAPK expression	Increase Smad3 and fibronectin expression Decreased Smad7 expression	DN model in Sprague Dawley rats induced by STZ	[86]
MAPK Signaling	Increase in p-ERK, ERK, p-p38 and INK expression	TGF-*β*1 activation and ECM deposition	UUO model of Renal fibrosis in Male C57BL/6 mice; HK-2 and NIH-3T3	[87]

TGF-*β*, transforming growth factor-*β*; Nox-4, nicotinamide adenine dinucleotide phosphate oxidase 4; Smad, suppressor of mother against decapentaplegic; C57BL/6, C57 black 6 strain of mice; p53, tumor protein 53; *i.p.,* intraperitoneal; BALB/c mice, Bagg Albino/c strain of mice; UUO, unilateral ureteral obstruction; YAP, Yes-associated protein; NUAK1, NUAK family kinase 1; *α*-SMA, alpha-smooth muscle actin; EMT, epithelial to mesenchymal transition; HO-1, heme-oxygenase 1; GPx4, glutathione peroxidase 4; Nrf-2, nuclear factor erythroid 2-related factor 2; ECM, excessive extracellular matrix; NF-κB, nuclear factor kappa-light-chain-enhancer of activated B cells; MAPK, mitogen-activated protein kinase; ERK, extracellular signal-regulated kinase; JNK, c-JUN N-terminal kinases; STAT3, signal transducer and activator of transcription; JAK, anus tyrosine kinase. UIRI, unilateral renal ischemia-reperfusion injury; CTGF, connective tissue growth factor; TXNIP, thioredoxin-interacting protein; NLRP3, nucleotide-binding leucine-rich repeat receptor family pyrin domain containing 3; SOD2, superoxide dismutase 2; ICR, institute of cancer research; PCNA, proliferating cell nuclear antigen; Bax, BCL2 associated X; CTGF, connective tissue growth factor.
